# A gaze into the void: Anticipatory saccades toward prevented events

**DOI:** 10.3758/s13414-025-03019-4

**Published:** 2025-02-26

**Authors:** Solveig Tonn, Janna Teigeler, Roland Pfister, Matthias Gamer

**Affiliations:** 1 Department of Psychology, Trier University, Johanniterufer 15, 54292 Trier, Germany; 2 Department of Psychology, University of Würzburg, Würzburg, Germany

**Keywords:** Prevention, Action representation, Anticipatory saccades, Monitoring

## Abstract

It is an open question how prevented events are represented in the human cognitive system—are they represented like produced events or are they represented in a different format? Here, we propose that seemingly contradictory observations on this question can be unified by assuming different time courses for production and prevention actions. Evidence from two experiments in the anticipatory saccades paradigm supported this model. Specifically, our results suggest that prevented events might be represented like produced events during action selection and execution, whereas their representation dissolves rapidly during action monitoring. In other words, the representation of prevented events reflects a two-step process: An initial affirmative representation is followed by later negation. Preregistrations, data, and analysis scripts for all experiments are available online (https://osf.io/m3veh/).

**Supplementary information:**

The online version contains supplementary material available at 10.3758/s13414-025-03019-4.

## Introduction

The human action repertoire encompasses a diverse range of behaviors. Categorizing these behaviors within a goal-centered framework reveals two complementary action types: event-producing actions and event-preventing actions. Although both action types are crucial for human action control, they differ substantially in the structure of their goals. Production actions, on the one hand, have the goal to generate an observable event in the agent’s environment. Prevention actions^1^, on the other hand, have the goal to keep an event from occurring. Thus, both action types differ based on whether they are characterized by the presence or absence of environmental events. This raises the question of how this difference influences the integration of respective events into action representations.

For production actions, research inspired by the ideomotor framework (Greenwald, 1970; James, 1890; Shin et al., 2010) has provided ample evidence suggesting that goals are essential components of action representations: Production actions are represented in terms of the events they produce and the anticipation of these events consistently influences the selection, execution, and monitoring of respective actions^2^ (e.g., Brown et al., 2022; Janczyk & Kunde, 2020; Kiesel & Hoffmann, 2004; Kunde, 2001; Kunde et al., 2004; Pfeuffer et al., 2016, Pfister, Pfeuffer et al., 2014b; Wirth et al., 2018). Further, research from the field of sense of agency shows that anticipated events also influence how an action is monitored and/or evaluated: Actions and self-caused events are not only explicitly attributed as a causal chain (Haggard, 2017; Moore et al., 2012; Moore & Obhi, 2012), but are also implicitly integrated into a common representation. Typically, this is demonstrated by the so-called temporal binding effect (Haggard et al., 2002; Schwarz et al., 2019; Suzuki et al., 2019), as measured by retrospective assessments of the temporal structure of an action-event episode. For production actions, the perceived time points of the action and the resulting event are consistently reported to be attracted to each other.

For prevention actions, however, the cognitive mechanisms are currently unclear, and previous research has arrived at seemingly conflicting interpretations: Ideomotor-inspired research proposed that prevention actions are represented comparably to production actions, suggesting that prevention actions are also represented in terms of the events they prevent. Here, experiments utilizing mouse-tracking and response durations showed that the anticipation of prevented and produced events influenced actions similarly (Tonn et al., 2023). That is, mouse trajectories were consistently attracted toward the anticipated location of produced and prevented events. Likewise, in key press tasks, the expected duration of events shaped the duration of key-press actions that were made to produce or prevent these events. These results indicated that whether an event is present or absent is not crucial for the representational structure of actions. Rather, the pure mental image of an absent event in prevention actions was sufficient to evoke the same control mechanisms as a present event in production actions. Further support for this idea is provided by biopsychological studies showing that autonomic changes in skin conductance, heart rate, and pupil dilation occur not only during the anticipation of an inevitable painful electric stimulation, but also in scenarios where a quick response can still prevent this stimulation (e.g., Rösler & Gamer, 2019; Stegmann et al., 2024).

Research on sense of agency, by contrast, did not yield evidence in favor of a common representational format of prevention and production actions. Rather, this line of research argued that fundamentally different representations underlie prevention and production actions, a conclusion that was based on multiple experiments that showed no influence of the prevented event on the perceptual illusion of temporal binding. Specifically, robust temporal binding effects were constantly observed for production actions, but they were completely absent for prevention actions (Pfister et al., 2021).

Results from different lines of research, thus, came to different conclusions for the question of how prevention actions are represented, and at first glance, these conclusions seem to be mutually exclusive. However, we propose that the results might be reconciled into a comprehensive model of action representation by carefully considering the specific methodologies used in the previous experiments.

One way to integrate the previous results is to consider that the representations of prevented events might be similar for early phases of action control (action selection and execution), while they are different for later phases of action control (action monitoring and/or evaluation). Whereas mouse-tracking and response durations target early phases of action selection and execution (e.g., Kunde, 2003; Pfister, Janczyk et al., 2014a, Pfister et al., 2023; Spivey & Dale, 2006; Wright et al., 2001), temporal binding targets the later phase of action evaluation (Haggard & Tsakiris, 2009; Majchrowicz & Wierzchoń, 2018; Vogel et al., 2021). This is particularly true because temporal binding is assessed retrospectively by asking participants about the perceived timing of actions and events only after the completion of the whole action-effect episode. Thus, prevented events might be represented similarly to produced events during early phases of an action, but this similarity in representations might only hold until the physical execution of an action is completed. Consequently, the representation of prevented events would be absent or reduced in later phases, such as action monitoring and evaluation.

However, it is also conceivable that the two action types are represented in the same way, even in later phases of action control. This speculation is supported by the currently emerging debate about the mechanisms underlying temporal binding. While it was previously assumed that this measure reflects a perception bias stemming from an implicit sense of agency for observed events (e.g., Beck et al., 2017; Borhani et al., 2017; Christensen et al., 2019; Haggard et al., 2002; Obhi & Hall, 2011), doubts about this interpretation arose during the past years (e.g., Kirsch et al., 2019; Schwarz et al., 2019; Siebertz & Jansen, 2022; Thanopoulos et al., 2018; Tonn et al., 2021). While alternative underlying mechanisms like multisensory integration have been suggested, they are still under debate (Gutzeit et al., 2023; Hon, 2023; Klaffehn et al., 2021, 2024; Lush et al., 2019; Schwarz & Weller, 2023; Tanaka, 2024). Therefore, a cautious interpretation of the currently available evidence suggests that while temporal binding most likely interacts with action monitoring and/or evaluation, it may not be the ideal approach for specifically investigating it. Consequently, it is not well-suited for drawing strong conclusions about the temporal evolution of action representations.

A more promising approach to investigate the representation of prevented events is the use of anticipatory eye movements as a direct marker of monitoring (Gouret & Pfeuffer, 2021b; Land, 2006; Land & Lee, 1994; Pelz & Canosa, 2001; Pfeuffer et al., 2016; Vig et al., 2011). Crucially, these saccades occur spontaneously and precede the onset of a visual event, thus reflecting the cognitive representation of a future event rather than a response or reflex triggered by an overt event. In typical setups that investigate action representations with anticipatory saccades, visual events are displayed at a specific position with a predefined delay after a response is made. These setups yield a systematic impact of anticipated visual events on different eye-movement parameters during the anticipatory time window, including the number of saccades toward the location of the future events, the position of the final fixation, and saccade latency (Pfeuffer et al., 2020). Eye movements, therefore, allow online access to action monitoring precisely as it occurs, in contrast to the retrospective evaluation obtained by temporal binding. The two experiments reported in the following took advantage of this highly sensitive measure to answer the question of whether prevented events are represented in action monitoring. While Experiment 1 used visual events of positive and negative valence to provide high external validity, Experiment 2 used visual events of neutral valence to provide high internal validity.

## Experiment 1

Experiment 1 used anticipatory eye movements to investigate the representation of produced and prevented events during action monitoring. The events were visual image stimuli that participants could produce or prevent by pressing a key. For production actions (i.e., actions that predictably generated a visual event), we expected to observe the typical pattern of anticipatory saccades toward the location of the future event. For prevention actions (i.e., actions that predictably kept a visual event from occurring), the number of anticipatory saccades provides crucial insights into the representation of prevented events: If anticipatory saccades toward prevented visual events occur comparably to anticipatory saccades toward produced visual events, this would suggest that both events are represented similarly during action monitoring. If, however, less anticipatory saccades are executed for prevention actions, this would suggest that the representation of prevented events is weaker during action monitoring. To cross-validate the conclusions derived from the number of anticipatory saccades, we also assessed the vertical position of anticipatory fixations. Again, comparable fixation positions for prevention actions and production actions would suggest similar representations during action monitoring, whereas fixation locations closer to the starting position for prevention actions would suggest weaker representations during action monitoring. Because eye movements often occur even in the absence of self-caused visual changes, we introduced an additional manipulation of whether the participants’ actions could influence a predetermined outcome (active trials) or not (passive trials). The passive trials served as baseline measurement against which the active trials were compared, thus allowing us to separate the effect of acting to make an event present/ absent from the effect of knowing that an upcoming event will be present/ absent. Specifically, we were interested in the comparison of active prevention versus passive production trials, as in both conditions, no picture was presented. However, this absence was either actively caused by the participant or predetermined.

### Methods

#### Transparency and openness

Preregistration, data, and analysis scripts are available on the Open Science Framework (https://osf.io/m3veh/). Slight deviations from this preregistration are explained in the Supplemental Material  and did not change the pattern of results. The research project was approved by the local ethics committee of the University of Wuerzburg (GZEK 2023-12).

#### Participants

Forty-eight volunteers participated in this experiment (age: *M* = 26.1 years, *SD* = 9.5; 37 women, 10 men, one nonbinary). They provided informed consent and received monetary compensation. The sample size was calculated based on effect-size estimates of previous work on anticipatory eye movements (Pfeuffer et al., 2016; Exp. 3: η_p_^2^ = .21) and yielded a high power of 1 − β = .90 for detecting anticipatory saccades toward future visual events. Eight participants were replaced because of more than 50% missing or outlier baseline measurements in eye-tracking (two participants), less than 50% correct keypress responses (five participants), or both (one participant).

#### Apparatus and stimuli

Participants sat in front of a 24-inch computer screen with a resolution of 1,920 px × 1,200 px and operated a standard keyboard. Viewing distance was 59 cm, and an EyeLink 1000 Plus (SR Research Ltd., Ottawa, Canada) system in the tower mount configuration was used to track the participants’ right eye at a sampling rate of 1000 Hz.

**Fig. 1:**
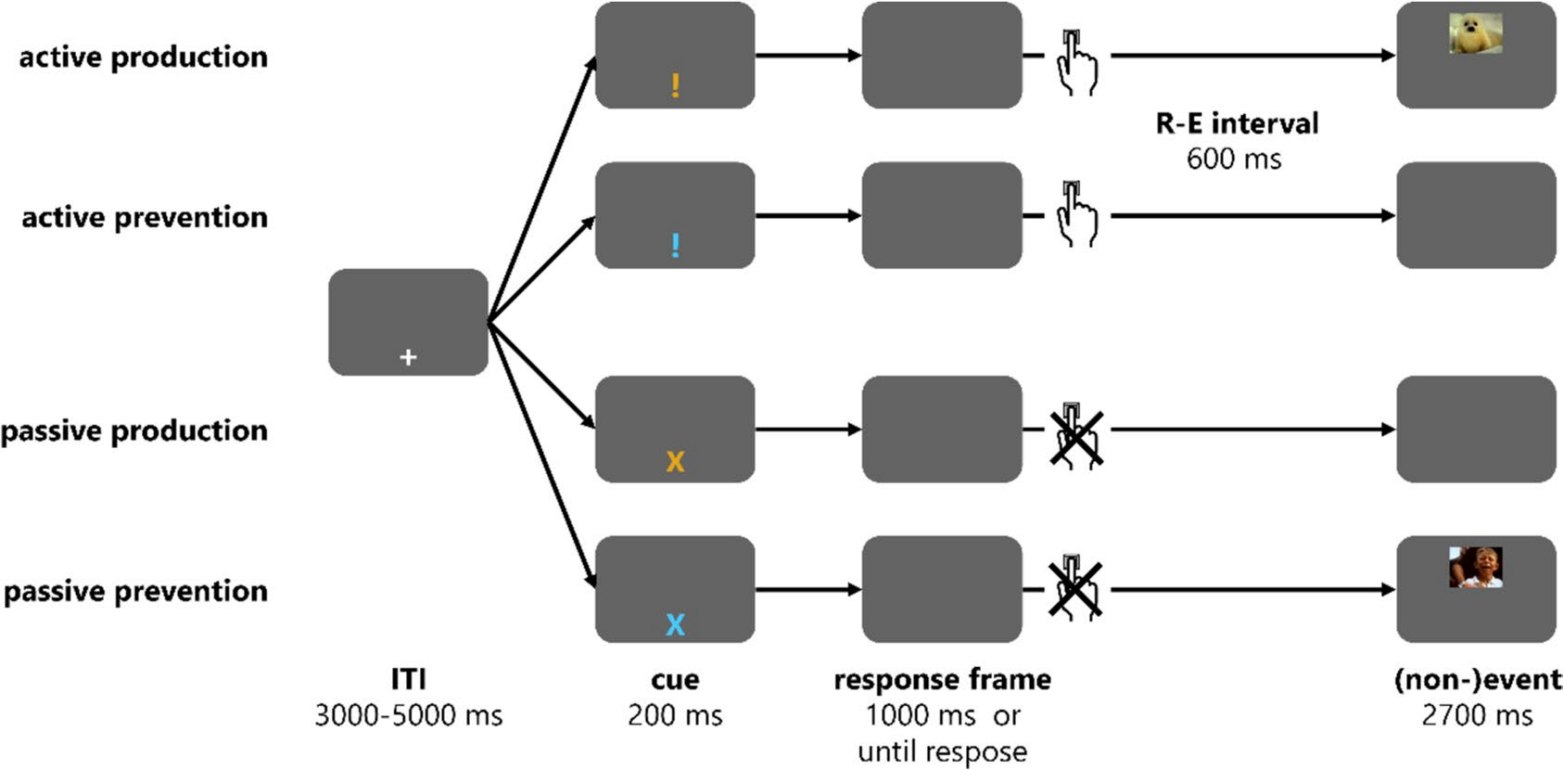
A 2 (action type) × 2 (controllability) within-subjects design was used in this experiment. All conditions were varied trial wise, and in each trial a cue announced the current condition: In active trials, participants could influence an upcoming event by pressing a key, but in passive trials, the upcoming event was predetermined. In active production trials, a key press was followed by a picture. In active prevention trials, a key press was followed by the absence of a picture. In passive production trials, the absence of a picture was predetermined (no production possible). In passive prevention, the presence of a picture was predetermined (no prevention possible). Active trials without key presses and passive trials with key presses were excluded as errors

All stimuli were displayed against a grey background. A white “+” was shown as fixation cross 150 px below the screen center. At the same location, cues (shape: “!” vs. “X” and color: blue vs. yellow) signaled the condition of the current trial (see Fig 1). The shape indicated the controllability of the current trial: While the exclamation mark “!” announced an active trial in which participants could influence events by pressing the space bar, the capital “X” announced a passive trial in which events were predetermined. Both cues could be blue or orange in color. This color announced the action type of the current trial—that is, whether participants could intentionally produce visual events on the screen (production trials) or could intentionally prevent visual events on the screen from occurring (prevention trials). The mapping of action types to colors was counterbalanced across participants.

Pictures (500 px × 350 px) from the International Affective Picture System (IAPS; Lang et al., 1997) served as visual events and were presented 575 px above the cue (center to center). To provide a realistic scenario for produced and prevented events, the picture set used in this experiment contained 100 pictures—50 of positive valence used for production actions (mean normative ratings: valence = 7.49, arousal = 5.04) and 50 of negative valence used for prevention actions (mean normative ratings: valence = 3.12, arousal = 5.24 mean).

#### Procedure

In the beginning of the experiment, participants were informed about the different conditions. For each condition it was explained which cue announced the condition, whether a key press was required, and whether a picture would be presented. Participants received no instructions regarding eye movements, except the instruction to fixate the fixation cross when it was presented. Detailed instructions are provided in the Supplemental Material.

After the instructions, participants were able to familiarize themselves with the design and explore the different stimulus-action-event mappings in a training block consisting of 20 trials. Before the actual experiment started, they could ask questions and again received the instructions to ensure complete understanding of the task. The actual experiment consisted of two blocks with 100 trials each. Prior to each block, the eye tracker was calibrated and validated using a nine-dot grid (mean accuracy = 0.37°, *SD* = 0.13°). Action type (production vs. prevention) as well as controllability (active vs. passive) were varied trial-wise.

A trial started with the presentation of a fixation cross for a randomized duration between 3,000 ms and 5,000 ms, and participants were instructed to fixate it. The fixation cross was followed by the presentation of the cue for 200 ms. With cue onset, a response frame started that ended with a key press response or after 1,000 ms if no key-press response was registered. Depending on the experimental condition, a picture was presented or not. The response-event interval between key press and picture presentation was 600 ms. Picture presentations lasted 2,700 ms; if no picture was presented, the screen remained blank for the same time.

Four different trial types were implemented (see Fig 1): In active production trials, a picture could be produced with a key press, and in active prevention trials, a picture could be prevented from occurring with a key press. In passive production trials, no picture was presented, and this could not be influenced by a key press. In passive prevention trials, a picture was presented, and this could not be influenced by a key press. Each data point can be described by three characteristics: The assignment to one action type (production or prevention), controllability (active = action; passive = no action), and event presentation (picture vs. no picture). Still, only two factors are necessary to describe all trial types as the third factor can be inferred from a combination of the other two. We decided to use action type and controllability for labelling the different conditions to stress how participants can change the environment by acting. Active trials comprised actions, and thus real production or prevention. Passive trials provided control conditions with outcomes that were identical to withholding an action in the respective active trials (i.e., the outcomes that occur in case of unsuccessful production or prevention). Pictures were presented in active production and passive prevention trials; no pictures were presented in passive production and active prevention.

After participants had completed both experimental blocks, they were asked to rate all pictures regarding their valence and arousal using a 9-point graphic Self-Assessment-Manikin (Morris, 1995) for each rating. Furthermore, participants filled out the Anxiety Sensitivity Index (ASI; Reiss et al., 1986; German version: Kemper et al., 2011), the Behavioral Inhibition System and Behavioral Activation System Scales (BIS-BAS Scales; Carver & White, 1994; German version: Strobel et al., 2001), the Sensitivity to Punishment and Sensitivity to Reward Questionnaire (SPSRQ; Torrubia et al., 2001; German version: Hewig et al., 2014), and the Intolerance of Uncertainty Scale (IUS; Freeston et al., 1994; German version: Gerlach et al., 2008). As preregistered, the questionnaires were solely collected for exploratory analyses that are not relevant for the current research question. Thus, results from these questionnaires are not reported here.

#### Data processing

Gaze coordinates were classified into saccades and fixations using EyeLink’s default criteria (saccades: eye movements exceeding a velocity of 30°/s or an acceleration of 8000°/s^2^; fixations: stable gaze between these saccades). For each trial, the last 300 ms before cue onset were defined as baseline. We then examined horizontal and vertical baseline positions separately for each participant and block to identify baseline outliers. Outliers were defined as trials where the baseline was more than 50 px away from the average baseline or where a recursive procedure marked them as outlier. Both criteria were computed independently. In the recursive procedure, we temporarily removed the highest and lowest value from the baseline coordinates before calculating the mean and standard deviation of the remaining distribution. If any of the removed values were more than three standard deviations from the mean of the remaining data, it was classified as outlier and permanently removed from the analysis. This procedure was repeated until no more outliers were detected (for further details on this procedure, see End & Gamer, 2017; Rösler & Gamer, 2019; Stegmann et al., 2024; van Selst & Jolicoeur, 1994). Trials with missing baseline or outlier baseline values (9.9%) were discarded from all further analyses. For all other trials, the baseline was used as offline correction for positional drift.

From the 600 ms before the visual (non-)event, we extracted the number of anticipatory saccades and the vertical position of the last fixation that was at least partially in the anticipatory interval. Anticipatory saccades were defined as saccades with an upward motion of more than 40 px (corresponding to 1° visual angle). Resulting number of trials that contained at least one anticipatory saccade were divided by the total number of valid trials in the respective condition to yield proportions. Vertical positions were defined as *y*-coordinate relative to the trial baseline, with positive values indicating upward direction. Pupil data were extracted for exploratory analyses.

Further, we extracted reaction times in case of key press responses (time between cue onset and key press) and error rates. Correct trials were defined as trials with a key press for active trials and without a key press for passive trials. Trials with incorrect responses (6.1%) were excluded from all analyses. Exploratory analyses regarding eye movements (i.e., reactive fixations and pupil size change), key presses, and valence and arousal ratings are available in the Supplemental Material.

#### Statistical analyses

Both dependent variables, proportion of anticipatory saccades and vertical position of the last fixation, were analyzed with a 2 (action type: prevention vs. production) × 2 (controllability: active vs. passive) within-subjects analysis of variance (ANOVA). Significant interactions were followed by *t*-tests, computed separately for each controllability condition. Further, we compared passive production trials against active prevention trials with a *t*-test, as in both trial types, no events were presented.

### Results

**Fig. 2:**
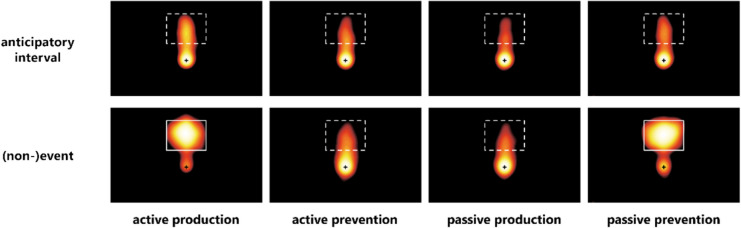
Fixation densities of Experiment 1 as a function of experimental condition and time interval. Color denotes probability density, with yellow indicating a high probability of fixations and black indicating a low probability of fixations. Dashed white rectangles indicate the area of potential picture presentation. Actual picture presentation is marked by solid lines. (Color figure online)

**Fig. 3:**
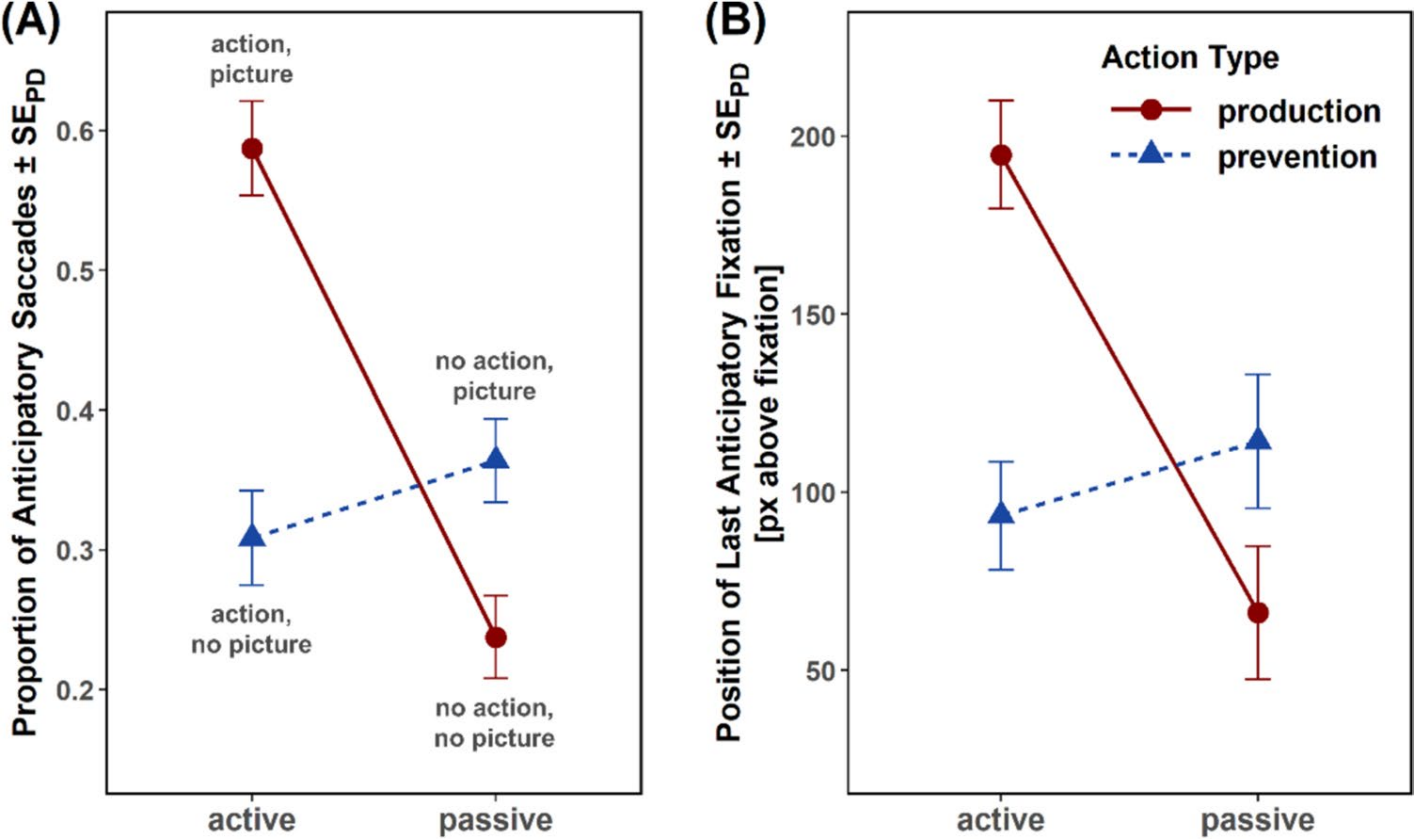
Results of Experiment 1. Mean proportion of anticipatory saccades (**A**) and mean y location of the last anticipatory fixation in pixels, with zero pixels denoting the location of the fixation cross (**B**) as a function of controllability (active vs. passive) and action type (production vs. prevention). Error bars represent standard errors of paired differences, computed separately for each comparison of production versus prevention conditions (Pfister & Janczyk, 2013)

Figure 2 illustrates the distribution of fixations of all experimental conditions in the anticipatory interval and the (non-)event phase. Results of the two main dependent variables, the proportion of anticipatory saccades and the vertical landing position of the last anticipatory saccade, are shown in Fig. 3.

#### Proportion of anticipatory saccades

Participants made more anticipatory saccades toward future visual events in production compared with prevention trials (41.3% vs. 33.6%), *F*(1,47) = 20.21, *p* < .001, η_p_^2^ = .30, and in active compared with passive trials (44.8% vs. 30.1%), *F*(1,47) = 50.26, *p* < .001, η_p_^2^ = .52. Action type and controllability interacted, *F*(1,47) = 56.42, *p* < .001, η_p_^2^ = .55, with more anticipatory saccades in active production than active prevention trials (58.8% vs. 30.9%), *t*(47) = 8.25, *p* < .001, *d* = 1.19, and a reversed difference between passive production and passive prevention trials (23.8% vs. 36.4%), *t*(47) = −4.24, *p* < .001, *d* = −0.61. Crucially, more anticipatory saccades were made in active prevention trials than in passive production trials (30.9% vs. 23.8%), *t*(47) = 2.71, *p* = .009, *d* = 0.39.

#### Vertical position of last fixation

The location of the last fixation within the anticipatory interval was higher in production compared with prevention trials (130 px vs. 104 px), *F*(1,47) = 4.94, *p* = .031, η_p_^2^ = .10, and in active compared with passive trials (144 px vs. 90 px), *F*(1,47) = 25.61, *p* < .001, η_p_^2^ = .35 (a *y*-coordinate of zero pixels denotes the location of the fixation cross, positive values indicate fixations above the fixation cross, and negative values indicate fixations below the fixation cross). Again, action type and controllability interacted, *F*(1,47) = 37.66, *p* < .001, η_p_^2^ = .44, with higher fixation positions in active production than active prevention trials (195 px vs. 93 px), *t*(47) = 6.67, *p* < .001, *d* = 0.96, and a reversed difference between passive production and passive prevention trials (66 px vs. 114), *t*(47) = −2.57, *p* = .014, *d* = −0.37. Crucially, the location was higher in active prevention than passive production trials (93 px vs. 66 px), *t*(47) = 2.29, *p* = .027, *d* = 0.33.

### Discussion

Experiment 1 showed that active prevention actions were accompanied by a significantly lower proportion of anticipatory saccades than active production actions. However, the number of anticipatory saccades in active prevention actions was still significantly higher than for passive production actions. Thus, anticipatory saccades occurred at a lower frequency when preventing a picture than when producing a picture. At the same time, more anticipatory saccades occurred when a picture was intentionally omitted than when the absence of a picture was predetermined. This pattern of results directly mirrors the distribution of last fixation locations within the anticipatory interval. Participants fixated a lower point on the screen when preventing a picture as compared with when producing a picture. At the same time, they fixated a higher point if they intentionally caused the omission of the picture compared with if the omission of the picture was the predetermined outcome. These observations suggest that prevented future events are represented during action monitoring in prevention actions. This representation, however, seems to be weaker than the representation of produced future events in production actions.

One possible alternative explanation for our findings stems from the valence of the pictures, because we had used positive pictures for production actions and negative pictures for prevention actions. This choice provides a naturalistic setting, as humans mostly produce events they perceive as positive and prevent events they perceive as negative. However, this design decision also comes with the limitation that the observed pattern of results might (in part) reflect a valence effect: Participants might not only prevent the perception of negative pictures by omitting the presentation of these pictures with their key presses. Rather, participants might additionally inhibit their eye movements to prevent the perception of negative picture when the visual event was not prevented successfully. To rule out this alternative explanation, Experiment 2 employed neutral pictures for both action types.

## Experiment 2

In Experiment 1, we showed reduced anticipatory saccades and lower fixations for prevention actions, compared with production actions. This could be explained either by differences in action representations or by the different picture categories that were used for these two action types (i.e., positive pictures in production trials, negative pictures in prevention trials). To disentangle these two possible influences, Experiment 2 controlled for valence by using neutral pictures for both action types.

### Methods

#### Transparency and openness

Preregistration, data, and analysis scripts are available on the Open Science Framework (https://osf.io/m3veh/). Slight deviations from this preregistration are explained in the Supplemental Material and did not change the pattern of results. This research was approved by the local ethics committee of the University of Wuerzburg.

#### Participants

Forty-eight new volunteers (age: *M* = 23.6 years, *SD* = 3.4; 33 women, 14 men, one diverse) participated in this experiment, provided informed consent, and received monetary compensation. Twenty-one participants were replaced because of more than 50% missing or outlier baselines in eye-tracking (one participant) or less than 50% correct key press responses (20 participants).^3^

#### Apparatus, stimuli, and procedure

Apparatus, stimuli, and procedure were the same as in Experiment 1 with only minor differences in the apparatus and one conceptual difference in the experimental manipulation.

While data of the first experiment was collected at the University of Wuerzburg, data collection of the second experiment was supported by PsychLab, a service of the Leibnitz Institute for Psychology (ZPID) in Trier. Here, participants sat in front of a 24-inch computer screen with a resolution of 1,920 px × 1,080 px instead of 1,920 px × 1,200 px. To maintain the same ratio of viewing angle to screen pixels as in Experiment 1, the screen to eye distance was set to 61 cm. Thus, the on-screen distances of all stimuli could remain identical. Further, the EyeLink 1000 Plus system was used in the desktop setup with a headrest instead of the tower mount configuration, and exploratory questionnaires were no longer collected. Again, the participants’ right eye was tracked and prior to each block, the eye-tracker was calibrated and validated using a nine-dot grid (mean accuracy = 0.48°, *SD* = 0.60°)

The crucial difference in the experimental manipulation was that instead of using pictures of different valences for production and prevention trials, 50 neutral IAPS pictures were used, each presented in both conditions (mean normative ratings: valence = 5.37, arousal = 3.20). This balanced the valence of the stimulus material in production and prevention conditions.

#### Data processing and statistical analyses

Data were treated and analyzed exactly as in Experiment 1. Again, trials with missing or outlier baseline (8.3%) and trials with incorrect responses (4.6%) were excluded from the analyses.

### Results

**Fig. 4:**
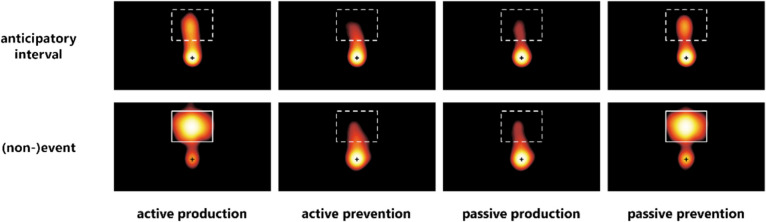
Fixation densities of Experiment 2 as a function of experimental condition and time interval. Color denotes probability density, with yellow indicating a high probability of fixations and black indicating a low probability of fixations. Dashed white rectangles indicate the area of potential picture presentation. Actual picture presentation is marked by solid lines. (Color figure online)

**Fig. 5:**
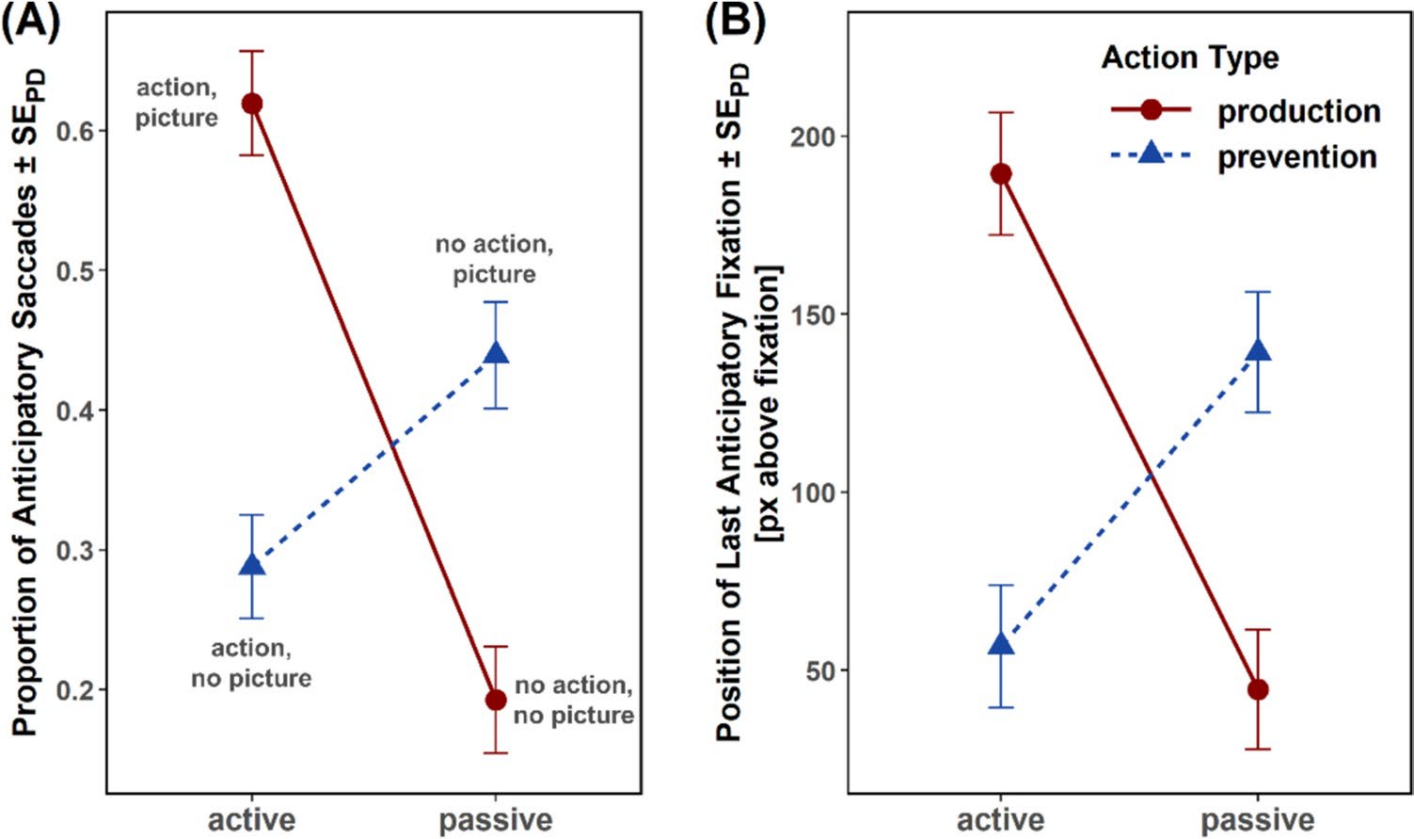
Results of Experiment 2. Mean proportion of anticipatory saccades (**A**) and mean y location of the last anticipatory fixation in pixel with zero pixels denoting the location of the fixation cross (**B**) as a function of controllability (active vs. passive) and action type (production vs. prevention). Error bars represent standard errors of paired differences, computed separately for each comparison of production versus prevention conditions (Pfister & Janczyk, 2013)

Figure 4 illustrates fixation heatmaps for all experimental conditions in the anticipatory interval and the (non-)event phase, whereas Fig. 5 visualizes the results for the two main dependent variables.

#### Proportion of anticipatory saccades

Participants made more anticipatory saccades toward future visual events in production compared with prevention trials (40.6% vs. 36.4%), *F*(1,47) = 10.72, *p* = .002, η_p_^2^ = .19, and in active compared with passive trials (45.4% vs. 31.6%), *F*(1,47) = 32.98, *p* < .001, η_p_^2^ = .41. Action type and controllability interacted, *F*(1,47) = 66.58, *p* < .001, η_p_^2^ = .59, with more anticipatory saccades in active production than active prevention trials (62.0% vs. 28.8%), *t*(47) = 8.89, *p* < .001, *d* = 1.28, and a reversed difference between passive production and passive prevention trials (19.3% vs. 43.9%), *t*(47) = −6.46, *p* < .001, *d* = −0.93. Crucially, more anticipatory saccades were made in active prevention than passive production trials (28.8% vs. 19.3%), *t*(47) = 3.59, *p* = .001, *d* = 0.52.

#### Vertical position of last fixation

The location of the last fixation within the anticipatory interval was higher in production compared with prevention trials (117 px vs. 98 px), *F*(1,47) = 13.47, *p* = .001, η_p_^2^ = .22, and active compared to passive trials (123 px vs. 92 px), *F*(1,47) = 13.33, *p* = .001, η_p_^2^ = .22. Again, action type and controllability interacted, *F*(1,47) = 49.27, *p* < .001, η_p_^2^ = .51, with higher fixation positions in active production than active prevention trials (189 px vs. 57 px), *t*(47) = 7.73, *p* < .001, *d* = 1.12, and a reversed difference between passive production and passive prevention trials (45 px vs. 139 px), *t*(47) = −4.25, *p* < .001, *d* = −0.61. Additionally, the location was descriptively, but non-significantly higher in active prevention trials than in passive production trials, *t*(47) = 1.52, *p* = .134, *d* = 0.22.

### Discussion

Experiment 2 replicated the results of Experiment 1 and confirmed that active prevention actions were accompanied by anticipatory eye movements. As in Experiment 1, the proportion was lower than for active production actions, but higher than for passive production actions. Thus, the reduced proportion of anticipatory eye movements in prevention actions did not originate from the negative valence of the pictures used in prevention trials of Experiment 1; rather, it indicates that while prevented events are indeed represented during action monitoring, this representation is considerably weaker than in production actions.

Although the main pattern of results was replicated, a subtle difference between experiments emerged when comparing the vertical position of the last fixation between active prevention and passive production actions: While this comparison was significant in Experiment 1, it resulted only in a descriptive difference in Experiment 2. Yet, in our primary measure, the proportion of anticipatory saccades, the difference between active prevention and passive production actions was highly significant in both experiments. Thus, the location of the last fixation could be a slightly less sensitive measure than the proportion of anticipatory saccades, at least when eye-tracking hardware with high temporal and spatial resolution is used.

## General discussion

The present study employed anticipatory eye movements to investigate action representations in prevention actions. Specifically, we aimed to reconcile previous contradictory results by answering the question whether prevented events are represented like produced events during action monitoring. This was motivated by evidence for similar representations during earlier phases of action selection and execution (Tonn et al., 2023) and evidence for absent representations during later, retrospective phases of action evaluation (Pfister et al., 2021). In two experiments, we consistently found that prevented events were still represented during action monitoring. This representation, however, was substantially weaker than the representation of produced events. Therefore, the current results provide a critical missing piece for a comprehensive model on the representation of prevention actions: Together with previous findings, our results suggest that prevented and produced events are represented similarly during action selection and execution, but after successful execution of the action, the representation of the prevented event dissolves faster than the representation of produced events. Therefore, this representation is reduced during (post-action, pre-event) monitoring and completely absent in retrospective (post-event) action evaluation.

The pattern proposed by this model mirrors the two-step model that is commonly assumed to underlie negation processing (e.g., Dudschig & Kaup, 2020; Gilbert, 1991; Gilbert et al., 1990; Kaup et al., 2006). Both models posit that agents initially form a representation of the to-be-negated or to-be-prevented event. Subsequently, this representation is negated. While both models clearly differentiate between these two subsequent steps, the timing of the negation or dissolution of this representation may differ. Unlike in classical negation studies (e.g., Jones, 1966; Just & Carpenter, 1971, 1976; Wason, 1959), the investigated prevention actions do not require a negation of the event representation prior to the selection of the correct action. Rather, the available empirical evidence suggests that for prevention actions, this dissolution occurs only after the correct action is executed and thus, during action monitoring. This mechanism presumably facilitates efficient resource allocation. In highly predictable environments, such as the current experimental setting, successful prevention is entirely contingent on successful action execution. Therefore, redirecting resources toward future tasks can be started once sufficient information about the action’s correctness has been acquired (e.g., through body-related events like the tactile or proprioceptive feedback from the key press; Pfister, 2019). Whether the representation of prevented events might persist to an even higher amount during action monitoring in less predictable environments, in which the success of the prevention action is less contingent on the correctness of an action, remains to be tested.

The reduced monitoring of prevented events also suggests that maintaining the representation of omitted events is difficult. The cognitive system in general has been shown to have difficulties in dealing with absent events (e.g., Newman et al., 1980; Treisman, 1986; but see Cochrane & Milliken, 2019) and this is in line with research from the field of visual search. Here, a robust prevalence effect has been demonstrated, indicating that targets are more frequently missed if they are unlikely to occur (Wolfe et al., 2005), and this effect even persists against efforts to increase monitoring (e.g., by instructing a cautious response strategy, providing participants the possibility to correct errors or explicitly prompting them to confirm their previous estimation; van Wert et al., 2009; Wolfe et al., 2007). This prevalence effect is challenging when executing tasks where events are unlikely, but important to detect, such as security personnel screening luggage or doctors detecting tumors (Horowitz, 2017; Mitroff & Biggs, 2014). Similarly, reduced monitoring, driven by an assumed absence of environmental events because of executed prevention actions, can have detrimental consequences. This is especially the case when there is an initial misjudgment of the relationship between an action and the omission of an environmental effect: If the presumed contingency is low, monitoring might be reduced prematurely. As a result, undetected events may occur, rendering both compensating actions as well as adjustments to the incorrect contingency assumptions impossible.

Perhaps surprisingly, however, there are also cases of overly strong monitoring in case of omitted events. Patients with anxiety disorder, for instance, show increased vigilance and error-monitoring across different tasks (Aarts & Pourtois, 2010; Armstrong & Olatunji, 2012). These patients usually also have severe problems performing on tasks requiring efficient cognitive processing (Berggren & Derakshan, 2013). The present findings suggest that performance issues may be a direct consequence of their prolonged monitoring, as this could be particularly taxing on the cognitive system.

The present observation of (limited) monitoring in prevention behavior further resembles a longstanding question in avoidance research: How can avoidance actions be reinforced if they are never followed by any environmental events as soon as the avoidance behavior is implemented? In avoidance research, both affective states and the knowledge about the omission of the avoided event have been identified as important determinants (Eder & Dignath, 2014; Krypotos et al., 2015; Lovibond, 2006; Mowrer, 1939; Seligman & Johnston, 1973). Here, our experiments further stress the role of avoided events by demonstrating that these events differentially enter action representations throughout all phases of an action, even in highly predictive settings where prevented events are hardly ever perceived.

Besides these theoretical contributions on the representational content and monitoring of prevention or avoidance actions, our orthogonal manipulation of action type (production vs. prevention) and controllability (active vs. passive) also allows for another comparison. That is, it allows us to assess how self-caused and environment-caused events are preceded by anticipatory eye movements. Previous research on anticipatory eye movements has shown that anticipatory saccades occur for environment-caused events (e.g., stimulus sequences; Haith et al., 1988; Land & Hayhoe, 2001; Land & Lee, 1994; Patla & Vickers, 1997) and that they occur for self-caused events (Gouret & Pfeuffer, 2021a; Pfeuffer et al., 2016, 2020). However, we are not aware of any study specifically investigating whether anticipatory eye movements are influenced by this locus of control. Construing the current results as the factorial combination of locus of control (self-caused vs. environment-caused) and event occurrence (picture presentation vs. no picture presentation) allows for this comparison: Here, our results provide promising evidence that self-caused (non-)events are preceded by more anticipatory eye movements than environment-caused (non-)events.^4^ This comparison is especially interesting from the perspective of sense-of-agency research. Within this line of research, there is ample evidence that the perception of self-caused events is distinctly different from the perception of environment-caused events (Blakemore et al., 1998; Farrer & Frith, 2002; Haggard et al., 2002; Waszak et al., 2012). Thus, our results provide the first empirical evidence for a previous speculation that anticipatory saccades might be related to the sense of agency (Pfeuffer et al., 2016). Whether this relation also holds in more complex settings or whether the observed data pattern can be explained by a general increase of activity upon action (be it manual activity or eye movement activity), remains to be tested.

Alternative explanations are also conceivable for the observed difference in anticipatory saccades between active prevention and passive production actions. Just as a general increase of activity upon action could account for the different number of anticipatory saccades when comparing actions with a different locus of control, a general increase of activity upon action could also account for the observed difference between these two conditions. This is the case as both trial types technically only differ in whether an action is executed or not. Further, anticipatory saccades in active prevention might be intentionally implemented to check whether the prevention action was successful, and the picture is indeed not presented. This would still speak for a representation of the prevented event during action monitoring, but the format of the representation would be more explicit, rather than implicit. While the overall low error rates and the equal distribution of anticipatory saccades throughout the experiment suggest that this is not the core mechanism underlying the observed pattern, we cannot fully preclude this possibility.

## Conclusion

In conclusion, the present research provides critical insights that enable a nuanced understanding of how prevented events are represented during different action phases: Whereas prevented events enter action selection and execution comparably to produced events, their representation is dissolved during monitoring and thus decays earlier than the representation of produced events. This marks a two-step process in the representation of prevented events—a first step, where the representation of this event is built up, and a second step, where the representation is negated and dissolved.

## Supplementary information

Below is the link to the electronic supplementary material.

Supplementary file1 (DOCX 333 KB)

## Data Availability

Both experiments were preregistered. Preregistrations, data, analyses, and figure scrips are publicly available on the Open Science Framework (https://osf.io/m3veh/).

## Footnotes

1 Production actions are also referred to as operant actions. Prevention actions are also referred to as active avoidance actions.

2 Here, we break down action-event episodes into four “phases”: Action selection, execution, monitoring, and evaluation. However, this terminology does not necessarily imply a strictly serial sequence of encapsulated mental processes. Rather, these phases may overlap in time and share information (see Tonn et al., 2024, for a discussion on the overlap of such phases in mouse tracking). Consequently, our use of “phase” does not refer to the “stage” concept in classic stage theory (Donders, 1969; Sternberg, 1969). Rather, it intends to establish approximate temporal and functional reference points within the course of an action.

3 With the neutral pictures used in Experiment 2, some participants were no longer motivated to prevent the pictures in the active prevention condition, presumably because experiencing sensation is more rewarding than being deprived of sensation (Eder et al., 2022; Wilson et al., 2014). This can account for the high participant dropout in Experiment 2.

4 Please note that in this specific case, no reanalyses are required because relabeling the conditions only relabels the resulting inferential statistics. While the main effect of controllability reflects the main effect of locus of cause, the interaction between action type and controllability reflects the main effect of event type, and the main effect of action type reflects the interaction between event type and locus of cause.
