# Supplementary material for: A gaze into the void: Anticipatory saccades toward prevented events
Source: Atten Percept Psychophys. 2025 Feb 26;87(3):848–61. doi: 10.3758/s13414-025-03019-4 (PMC11965180; doi:10.3758/s13414-025-03019-4)
Supplement: Supplementary file 1 — Supplementary file1 (DOCX 333 KB) [file 13414_2025_3019_MOESM1_ESM.docx]

Supplemental Material

[Supplemental Material 1](#_Toc185146091)

[Instructions 2](#_Toc185146092)

[Detailed Instructions 2](#_Toc185146093)

[Instruction summary 2](#_Toc185146094)

[Manual responses 3](#_Toc185146095)

[Methods 3](#_Toc185146096)

[Results 3](#_Toc185146097)

[Discussion 4](#_Toc185146098)

[Reactive fixations of presented visual events 4](#_Toc185146099)

[Methods 4](#_Toc185146100)

[Results 5](#_Toc185146101)

[Discussion 5](#_Toc185146102)

[Pupil data 5](#_Toc185146103)

[Methods 6](#_Toc185146104)

[Results 6](#_Toc185146105)

[Discussion 7](#_Toc185146106)

[Valence ratings 8](#_Toc185146107)

[Methods 8](#_Toc185146108)

[Results 9](#_Toc185146109)

[Discussion 9](#_Toc185146110)

[Deviations from the preregistered analysis plan 9](#_Toc185146111)

[Data exclusions 9](#_Toc185146112)

[Data preprocessing 10](#_Toc185146113)

[Vertical position 10](#_Toc185146114)

[Experiment 1, analyzed as preregistered 11](#_Toc185146115)

[Experiment 2, analyzed as preregistered 12](#_Toc185146116)

Instructions

Here we provide the instructions participants received in the beginning of the experiment. Instructions are presented exemplary for one counterbalancing group in Experiment 2. Color assignments varied between participants and picture valences varied between experiments. Please note that instructions were originally presented in German. The English instructions here are, thus, not a literal reproduction of the original instructions, but a close translation. Instructions always comprised one slide with detailed instructions, followed by another slide summarizing the key points.

Detailed Instructions

At the beginning of each run, you will see a fixation cross in the lower half of the screen. Please direct your gaze to this fixation cross as soon as it appears. Next, you will see a colored symbol. The symbol’s shape tells you whether you are in an active or passive run. If the shape is a "!", then you are in an active run, if the shape is an "X" then you are in a passive run. In an active run, you can press the space bar to influence what happens on the screen. In a passive run, you cannot press any key and can simply observe what happens. The color of the symbol tells you whether you can produce or prevent an image. If the symbol is colored blue, you can create a neutral image in the upper half of the screen in the active run if you press the space bar after the symbol has disappeared. If you do not press the space bar or if you are in a passive run, no image will be shown. If the symbol is colored yellow, you can prevent a neutral image from appearing in the upper half of the screen in the active run. If you do not press the space bar after the symbol has disappeared or if you are in a passive run, the image will be displayed. Continue with the space bar.

Instruction summary

! : Active picture production: If you press the space bar, you can produce a picture in the upper half of the screen; if you do not press the space bar, nothing happens.

X : Passive picture production: You cannot press the space bar, no picture is presented.

! : Active picture prevention: If you press the space bar, you can prevent a picture in the upper half of the screen; if you do not press the space bar, this picture is presented

X : Passive picture prevention: You cannot press the space bar, a picture is presented.

Please press the space bar, if you are ready to start the experiment.

Manual responses

There are two (not necessarily mutually exclusive) hypotheses about how our experimental manipulations should impact the ease of manual responses (as manifested in response times and error rates). First, participants might be more motivated to prevent negative pictures than to produce positive pictures (Kahneman & Tversky, 2013; McGraw et al., 2010; Tversky & Kahneman, 1991), leading to shorter response times and fewer errors in preventing actions compared to producing actions in Experiment 1. Second, building up a to-be-negated cognitive representation might be more effortful than building up a purely affirmative cognitive representation. Correspondingly, although finalizing the negation may not be necessary prior to action selection, preparing a to-be-negated representation could lead to longer reaction times and more errors in preventing actions compared to producing actions in both experiments.

Methods

Manual responses were compared regarding their reaction time (time between cue onset and keypress) and success rate, with correct trials comprising keypresses in active trials and no keypresses in passive trials. Please note that although participants were instructed to press a key in the active condition and to not press a key in the passive condition, they nevertheless had the choice of whether to follow this recommendation and further, no error feedback was provided if this recommendation was not followed.

Results

For Experiment 1, responses were faster in active production trials than in active prevention trials (402 ms vs. 425 ms), *t*(47) = 2.75, *p* = .008, *d* = 0.40. Further, less errors were made in active production trials compared to active prevention trials (4.5% vs. 13.5%), *t*(47) = 4.31, *p* < .001, *d* = 0.62.

The same held true for Experiment 2, with faster responses in active production trials than in active prevention trials (356 ms vs. 374 ms), *t*(47) = 2.69, *p* = .010, *d* = 0.39, and fewer errors in active production trials compared to active prevention trials (3.2% vs. 9.2%), *t*(47) = 3.95, *p* < .001, *d* = 0.57.

Discussion

Reaction times and error rates were consistently lower in production than in prevention trials across both experiments. Thus, the results might be seen as a tentative hint that the effort that is associated with building up a to-be negated representation could outweigh the potential motivational influence associated with preventing events that are perceived as negative.

Reactive fixations of presented visual events

In the current design, participants could prevent the perception of visual events not only by preventing the visual events through manual responses, but also by avoiding the perception of present events through averted gaze. If avoidance by gaze is indeed used to prevent negative stimulation, this strategy should also influence fixation times after the onset of the visual event. Specifically, during picture presentation, negative pictures should be fixated less than positive pictures in Experiment 1.

Methods

In contrast to the analyses reported in the main body of the manuscript, this analysis focused on eye-tracking data *after* the anticipatory interval, during the visual event itself. Specifically, for the time interval during the presentation of the positive and negative pictures in Experiment 1 or neutral pictures in Experiment 2, we computed the percentage of the time where fixations were inside (as opposed to outside) the bounding box of the pictures and compared these percentages via *t*-test.

Results

For Experiment 1, pictures with positive valence (i.e., those presented in the production condition) were fixated more than negative pictures (i.e., those presented in the prevention condition; 70.9% vs. 61.8%), *t*(47) = 5.39, *p* < .001, *d* = 0.78.

For Experiment 2, neutral pictures in the production condition were still fixated more than neutral pictures on prevention trials (63.0% vs. 61.3%), *t*(47) = 2.22, *p* = .031, *d* = 0.32.

Discussion

For Experiment 1, pictures with positive valence were fixated more than those with negative valence during their presentation. This suggests that avoidance by gaze was indeed used as strategy to prevent negative stimulation, and consequently, that negative pictures were indeed perceived as aversive. However, we still observed a small effect of action type on fixation times in Experiment 2. This indicates that a share of the systematic variance could also be related to slight differences in the average novelty of the pictures based on the complementary structure of both action types: While pictures in the production condition (positive pictures in Exp. 1) were displayed at most once (they were never displayed in the passive condition), pictures in the prevention condition (negative pictures in Exp. 1) were displayed at least once (they were always displayed in the passive condition). Thus, prevention pictures were slightly less novel than production pictures even in case of same pictures for both conditions, which might contribute to the lower fixation times.

Pupil data

We exploratively investigated pupillary responses as there are two opposing predictions on how the anticipation of the visual event might influence pupil dilations. First, the anticipation of an image, regardless of its valence, might lead to a constriction via an anticipatory pupillary light response (Mathôt & van der Stigchel, 2015). Alternatively, the anticipation of an image might be dependent on the anticipated arousal, with higher arousal leading to a dilation of the pupil (e.g., Snowden et al., 2016).

Methods

Data were down-sampled to 100 Hz and blink periods were linearly interpolated. Afterwards, data were low-pass filtered with a cutoff frequency of 2 Hz, converted to mm (Hayes & Petrov, 2016), and baseline-corrected with respect to the last 100 ms preceding cue onset. Finally, pupillary responses were quantified as the mean change in pupil diameter during the 600 ms anticipatory interval.

Results

For Experiment 1, participants had a descriptively, but non-significantly weaker pupil responses in production compared to prevention trials (0.019 mm vs. 0.007 mm), *F*(1, 47) = 3.53, *p* = .067, η_p_^2^ = .07. Further, pupillary responses were stronger in active compared to passive trials (0.064 mm vs. -0.038 mm), *F*(1, 47) = 339.92, *p* < .001, η_p_^2^ = .88. Action type and controllability did not significantly interact, *F*(1, 47) = 2.75, *p* = .104, η_p_^2^ = .06. Nonetheless, we observed stronger pupil responses in the passive prevention as compared to passive production trials (-0.003 mm vs. -0.005 mm), *t*(47) = -2.11, *p* = .040, *d* = -0.31, but active production and active prevention trials did not differ, *t* < 1.

For Experiment 2, action type did not influence pupil responses, *F*(1, 47) = 1.09, *p* = .301, η_p_^2^ = .02, but they were again stronger in active compared to passive trials (0.071 mm vs. -0.034 mm), *F*(1, 47) = 194.51, *p* < .001, η_p_^2^ = .81. Action type and controllability interacted, *F*(1, 47) = 6.49, *p* = .014, η_p_^2^ = .12., with weaker pupillary responses for active production than active prevention trials, *t*(47) = -2.82, *p* = .007, *d* = -0.41, and no difference between passive production and passive prevention trials, *t* < 1.


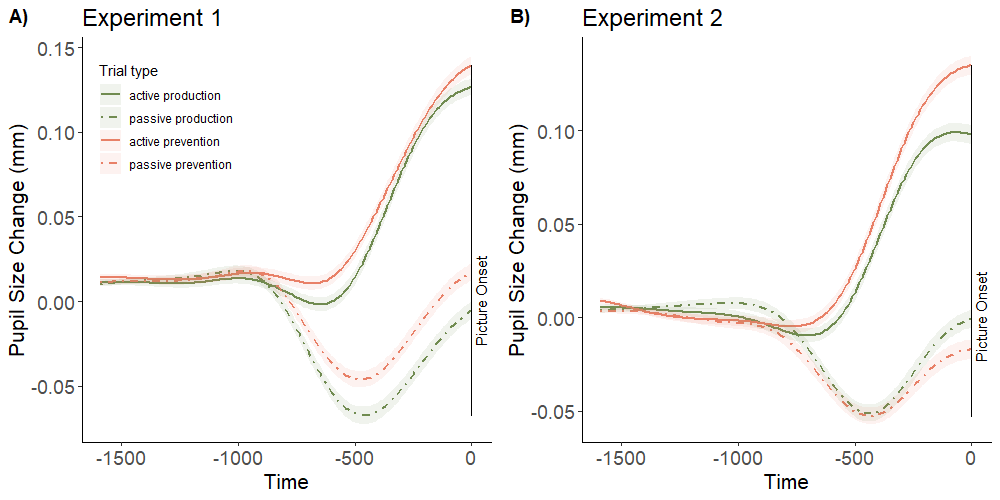


**Figure S1. Pupil size change in mm as a function of trial time relative to picture onset.** Confidence bands indicate standard errors of the means (SE).

Discussion

For both experiments, stronger pupillary dilation was observed in active as compared to passive trials. This result aligns with existing research that found systematic co-variation between pupil dilation and motor actions, conceivably stemming from motor preparation and execution processes (e.g., Hupé et al., 2009; Kloosterman et al., 2015).

In Experiment 1, where the arousal of the images systematically varied with the action type (see section on Valence Ratings), we found a descriptively stronger pupil dilation in prevention trials than in production trials. This is in line with the slightly higher mean arousal of the pictures in the prevention conditions, as the anticipation of arousing stimuli has been shown to reliably induce strong pupil dilation (e.g., Snowden et al., 2016). This notion is further supported by the finding of stronger dilation in passive prevention trials than in passive production trials, which can be interpreted as the anticipation of arousing content as compared to the anticipation of the lack of change, which is arguably not very arousing.

In line with this interpretation, the difference between production and prevention trials vanished in Experiment 2 where the same stimuli were used for both action types. Interestingly, we observed a stronger dilation in active prevention than in active production trials in this experiment. This could potentially reflect higher task effort (van der Wel & van Steenbergen, 2018) or the lack of an anticipated pupillary light reflex (Mathôt & van der Stigchel, 2015), as picture were presented in the active production condition, but not in the active prevention condition. However, the exact origin of this effect needs to be investigated by targeted future experiments.

Valence and arousal ratings

The crucial difference between Experiment 1 and Experiment 2 is that in the former, the valence of the employed stimulus material for the visual events differed for production and prevention conditions. While pictures were selected based on the normative IAPS picture ratings, such evaluations can be driven by personal aspects and might change over time. Thus, we cross-validated the intended valence manipulation by asking participants to rate the pictures at the end of the experiment.

Methods

Participants were asked to rate all pictures regarding their valence and arousal with a 9-point graphic Self-Assessment-Manikin (Figure S1), ranging from 1 to 9. Participants indicated their assessment with the number keys on the keyboard.


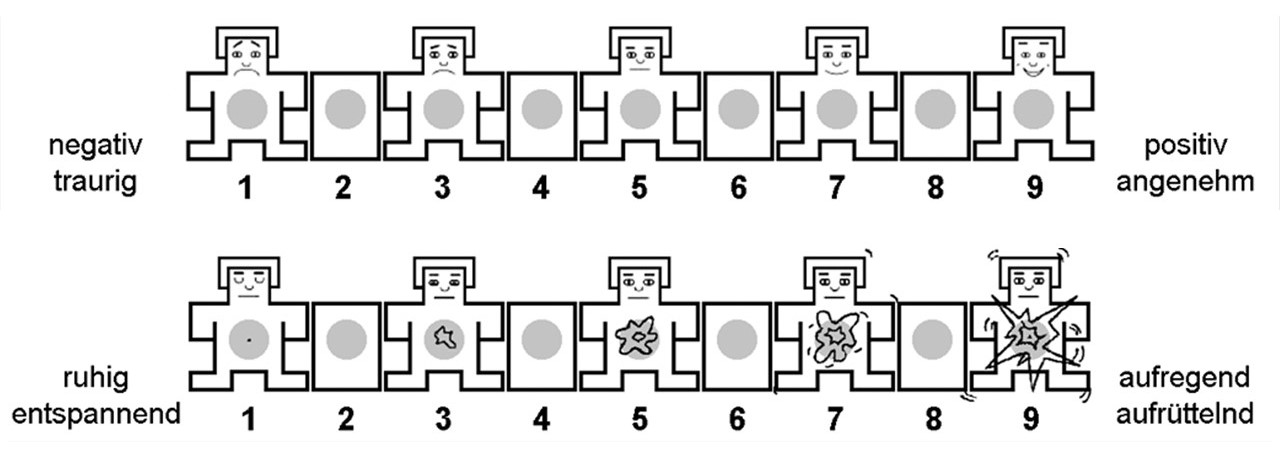


**Figure S2. 9-point graphic Self-Assessment-Manikin used for the valence and arousal ratings.** The upper manikin was used for the valence rating and ranged from the German words for negative/sad (1) to the German words for positive/pleasant (9). The lower manikin was used for the arousal ratings and ranged from the German words for calm/relaxing (1) to the German words for exciting/arousing (9).

Results

For Experiment 1, negative pictures were rated as more negative than positive pictures (2.70 vs. 7.60), *t*(45) = 26.37, *p* < .001, *d* = 3.89. In contrast to the original ratings, participants indicated that negative pictures were more arousing than positive pictures (6.09 vs. 3.15), *t*(45) = 12.55, *p* < .001, *d* = 1.85.

For Experiment 2, the same neutral pictures were used in the production and prevention condition. We nonetheless collected ratings of the employed pictures for completeness; the neutral pictures were rated with a mean valence of 5.64 and a mean arousal of 3.87.

Discussion

Participants indeed perceived negative pictures as more negative than positive pictures. Interestingly, the negative pictures were also rated as more arousing than the positive pictures, although they were matched based on the normative ratings. However, this does not weaken, but rather strengthen the current results: To-be-prevented, negative visual events were represented less than to-be-produced, positive events, although the negative events were more arousing and thus, conceivably more salient.

Deviations from the preregistered analysis plan

As reported in the main text, the final analysis procedure contains slight deviations from our preregistrations. In the current section, we explain the rationale behind these changes and report an analysis of the data with the preregistered procedure. Please note that none of the deviations from the preregistered analyses changed the pattern of results. Thus, this section does not provide novel insights, but rather aims at providing transparency on how we obtained our results.

Data exclusions

As preregistered, outliers were defined as trials where a recursive procedure with the cutoff set to 3 *SD*s classified the baseline as outlier. Yet, and in contrast to the preregistration, we additionally regarded trials where the baseline was more than 50 px away from the average baseline as outliers. We realized that we forgot to include this detail in the preregistrations only after data for both experiments were collected. Because we think that this criterion improves the quality of eye-tracking data by detecting participants that consistently do not fixate the fixation cross at the beginning of the trial, we decided to report the results with this additional outlier criterion. Please note that this affected the participant selection, leading to the replacement of two participants in Experiment 1 and one participant in Experiment 2.

Data preprocessing

In Experiment 1, we did not precisely specify how saccades would be extracted from the raw eye tracking data. While the most conservative approach would be to use the default EyeLink classification (velocity and acceleration criterion), we opted for an additional implementation of a motion magnitude criterion of 1° of visual angle, thus using a similar saccade classification as previous research on anticipatory saccades (Pfeuffer et al., 2016). While this has a large impact on the absolute number of anticipatory saccades detected, the relative number of saccades (i.e., the between-condition differences) are virtually unaffected by this choice. For completeness, we will report the saccade analyses for both experiments with both cutoffs here.

Vertical position

In Experiment 1, we preregistered to analyze the vertical end point of the first anticipatory saccade to investigate whether participants direct their gaze to the exact location of the anticipated visual event. At first glance, the end position of a saccade (the preregistered dependent variable) corresponds to the position of the following fixation (the reported dependent variable), and thus, the choice to use fixation positions instead of saccade endpoints seems arbitrary. Yet, we realized that saccades (as classified by the EyeLink default algorithm) do not always occur as one smooth movement from cue to picture location but rather, can be fragmented into several shorter saccades. Furthermore, using endpoints of saccades necessitates the existence of such anticipatory saccades, leading to imprecise estimates or even missing data in experimental conditions with too few anticipatory saccades (specifically in the passive production condition). In sum, we realized that the position of the last fixation is a methodologically superior indicator for our theoretical question, compared to the end position of the first saccade. Please note that switching from saccades to fixations also required switching from the first saccade to last fixation of the anticipatory interval: Because fixations are defined as stable gaze between saccades, a potential saccade within the anticipatory interval is preceded and followed by a fixation. To measure a similar compound as with the end point of the anticipatory saccades, the location of the subsequent, final fixation has to be considered.

Experiment 1, analyzed as preregistered

Using the criteria of the preregistration, we would not have excluded two participants and thus, would not have collected data from two additional participants. Consequently, results are reported without these additionally collected participants. Trials with missing or outlier baseline (6.3%) and trials with incorrect responses (6.8%) were excluded from the analyses.

When not using the 1° cutoff, participants made more anticipatory saccades towards future visual events in production compared to prevention trials (60.0% vs. 55.3%), *F*(1, 47) = 8.46, *p* = .006, η_p_^2^ = .15, and in active compared to passive trials (64.4% vs. 50.9%), *F*(1, 47) = 52.47, *p* < .001, η_p_^2^ = .53. Action type and controllability interacted, *F*(1, 47) = 57.11, *p* < .001, η_p_^2^ = .55, with more anticipatory saccades in active production than active prevention trials (74.3% vs. 54.6%), *t*(47) = 7.78, *p* < .001, *d* = 1.12, and a reversed difference between passive production and passive prevention trials (45.8% vs. 56.1%), *t*(47) = -3.97, *p* < .001, *d* = -0.57. Additionally, more anticipatory saccades were made in active prevention trials than in passive production trials (54.6% vs. 45.8%), *t*(47) = 3.44, *p* = .001, *d* = 0.50.

When using the 1° cutoff, participants made more anticipatory saccades towards future visual events in production compared to prevention trials (40.7% vs. 33.6%), *F*(1, 47) = 18.68, *p* < .001, η_p_^2^ = .28, and in active compared to passive trials (44.6% vs. 29.6%), *F*(1, 47) = 49.93, *p* < .001, η_p_^2^ = .52. Action type and controllability interacted, *F*(1, 47) = 59.97, *p* < .001, η_p_^2^ = .56, with more anticipatory saccades in active production than active prevention trials (58.0% vs. 31.2%), *t*(47) = 8.57, *p* < .001, *d* = 1.24, and a reversed difference between passive production and passive prevention trials (23.4% vs. 35.9%), *t*(47) = -4.30, *p* < .001, *d* = -0.62. Additionally, more anticipatory saccades were made in active prevention trials than in passive production trials (30.9% vs. 23.8%), *t*(47) = 3.00, *p* = .004, *d* = 0.43.

The position of the last fixation within the anticipatory interval was higher in production compared to prevention trials (132 px vs. 102 px), *F*(1, 47) = 6.36, *p* = .015, η_p_^2^ = .12, and active compared to passive trials (140 px vs. 94 px), *F*(1, 47) = 23.65, *p* < .001, η_p_^2^ = .33. Again, action type and controllability interacted, *F*(1, 47) = 37.12, *p* < .001, η_p_^2^ = .44, with higher fixation positions in active production than active prevention trials (191 px vs. 90 px), *t*(47) = 6.58, *p* < .001, *d* = 0.95, and a reversed difference between passive production and passive prevention trials (73 px vs. 114 px), *t*(47) = -2.35, *p* = .023, *d* = -0.34. Additionally, the location was descriptively, but not significantly higher in active prevention trials than in passive production trials (90 px vs. 73 px), *t*(47) = 1.81, *p* = .076, *d* = 0.26.

Experiment 2, analyzed as preregistered

Using the criteria of the preregistration, we would not have excluded one participant and thus, would not have collected data from one additional participant. Consequently, results are reported without this additionally collected participant. Trials with missing or outlier baseline (7.3%) and trials with incorrect responses (6.5%) were excluded from the analyses.

When not using the 1° cutoff, participants made more anticipatory saccades towards future visual events in production compared to prevention trials (56.9% vs. 53.4%), *F*(1, 47) = 7.30, *p* = .010, η_p_^2^ = .13, and in active compared to passive trials (61.8% vs. 48.5%), *F*(1, 47) = 35.74, *p* < .001, η_p_^2^ = .43. Action type and controllability interacted, *F*(1, 47) = 62.46, *p* < .001, η_p_^2^ = .57, with more anticipatory saccades in active production than active prevention trials (74.8% vs. 48.8%), *t*(47) = 8.34, *p* < .001, *d* = 1.20, and a reversed difference between passive production and passive prevention trials (38.9% vs. 58.1%), *t*(47) = -6.11, *p* < .001, *d* = -0.88. Additionally, more anticipatory saccades were made in active prevention than passive production trials (48.8% vs. 38.9%), *t*(47) = 3.53, *p* = .001, *d* = 0.51, although in neither trial type, pictures were presented.

When using the 1° cutoff, participants made more anticipatory saccades towards future visual events in production compared to prevention trials (40.3% vs. 36.4%), *F*(1, 47) =9.21, *p* = .004, η_p_^2^ = .16, and in active compared to passive trials (45.6% vs. 31.1%), *F*(1, 47) = 36.86, *p* < .001, η_p_^2^ = .44. Action type and controllability interacted, *F*(1, 47) = 64.12, *p* < .001, η_p_^2^ = .58, with more anticipatory saccades in active production than active prevention trials (61.9% vs. 29.4%), *t*(47) = 8.56, *p* < .001, *d* = 1.24, and a reversed difference between passive production and passive prevention trials (18.8% vs. 43.5%), *t*(47) = -6.50, *p* < .001, *d* = -0.94. Additionally, more anticipatory saccades were made in active prevention than passive production trials (29.4% vs. 18.8%), *t*(47) = 3.99, *p* < .001, *d* = 0.58, although in neither trial type, pictures were presented.

The position of the last fixation within the anticipatory interval was higher in production compared to prevention trials (119 px vs. 100 px), *F*(1, 47) = 13.89, *p* = .001, η_p_^2^ = .23, and active compared to passive trials (126 px vs. 92 px), *F*(1, 47) = 15.66, *p* < .001, η_p_^2^ = .25. Again, action type and controllability interacted, *F*(1, 47) = 46.99, *p* < .001, η_p_^2^ = .50, with higher fixation positions in active production than active prevention trials (192 px vs. 60 px), *t*(47) = 7.61, *p* < .001, *d* = 1.10, and a reversed difference between passive operant and passive prevention trials (45 px vs. 140 px), *t*(47) = -5.51, *p* < .001, *d* = -0.79. Additionally, the location was descriptively higher in active prevention trials than in passive production trials (60 px vs. 43 px), *t*(47) = 1.91, *p* = .063, *d* = 0.28.
